# Supplementary material for: Monocytes undergo multi-step differentiation in mice during oral infection by Toxoplasma gondii
Source: Commun Biol. 2019 Dec 18;2:472. doi: 10.1038/s42003-019-0718-6 (PMC6920430; doi:10.1038/s42003-019-0718-6)
Supplement: Supplementary file 1 — Supplementary Information [file 42003_2019_718_MOESM1_ESM.pdf]

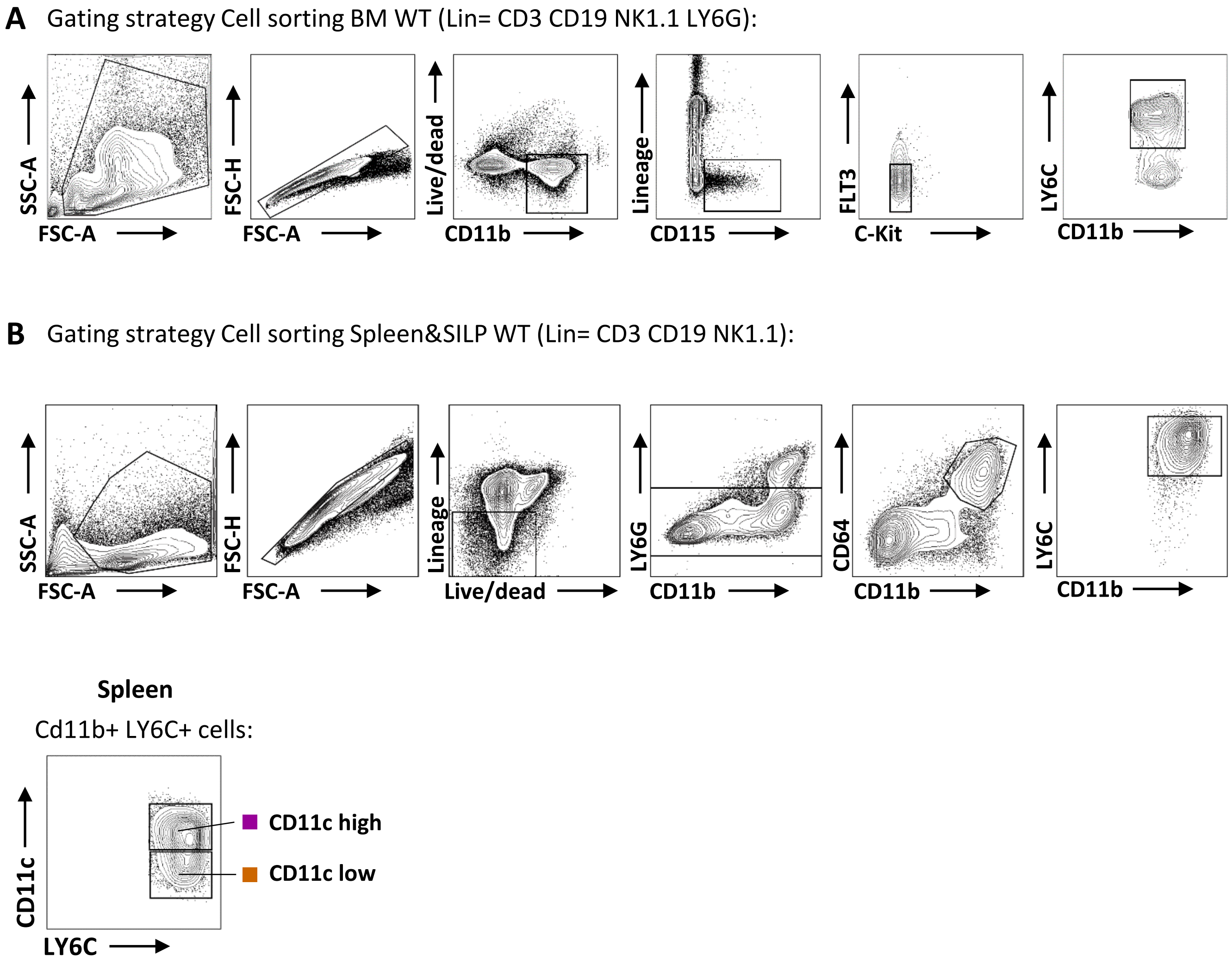

Supplementary figure 1: Gating strategy used to sort the Ly6C^{+} monocytes from the bone marrow (A), the SILP and the spleen (B).

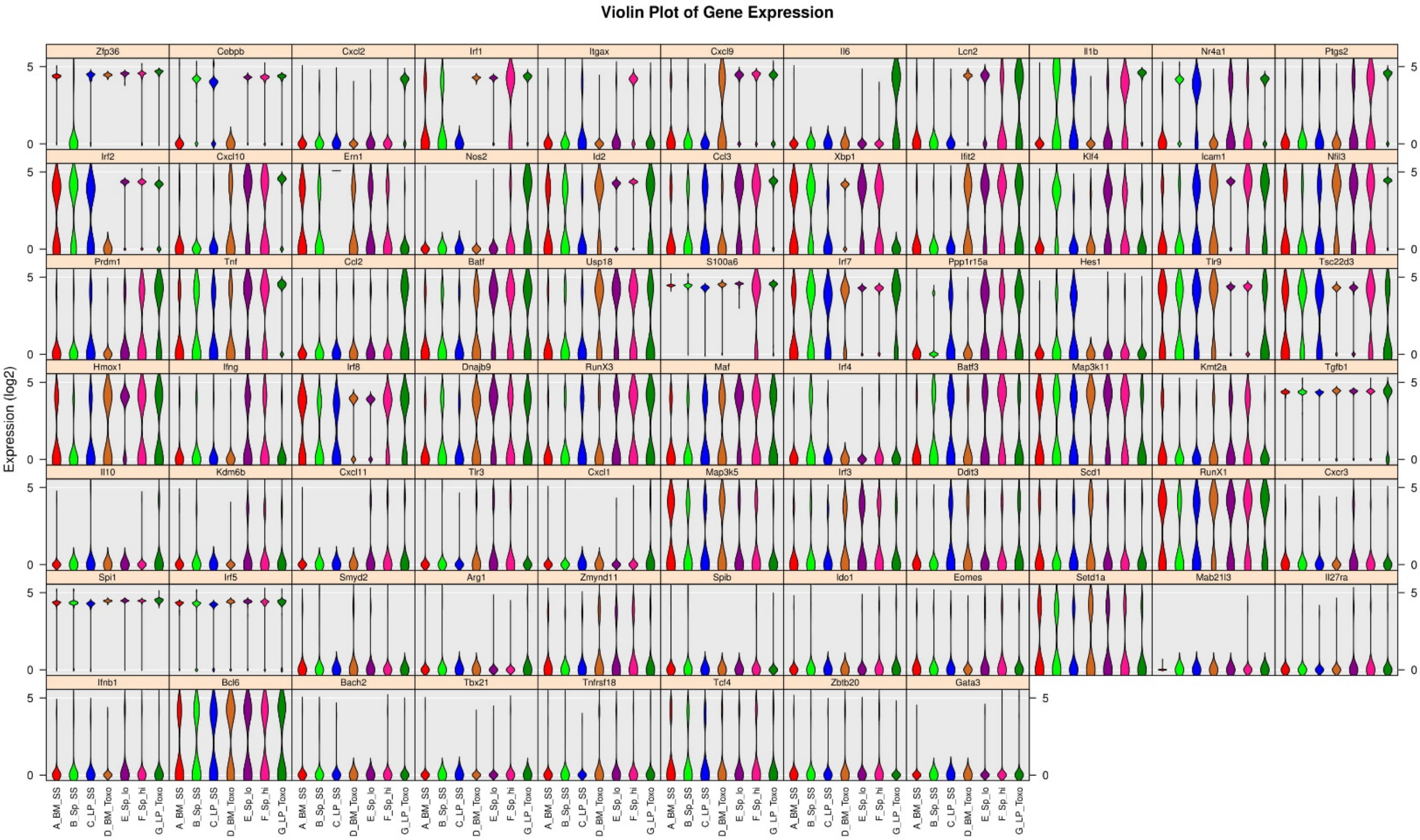

Supplementary figure 2: Violin plots representing scRTqPCR data in the monocytes from the BM, spleen (CD11c^{lo} or CD11c^{hi}) and SILP from steady-state and/or infected mice. The graphs are ordered according to the ANOVA p values.

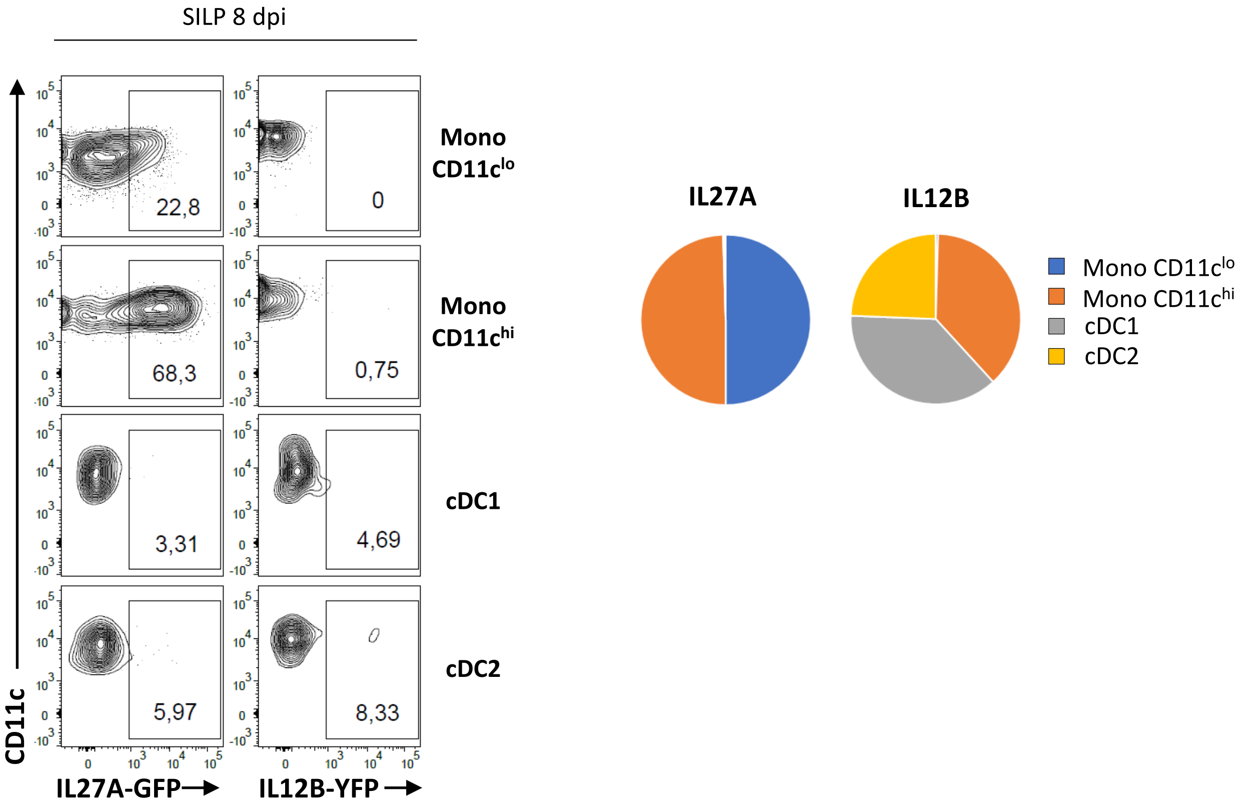

Supplementary figure 3: Representative flow cytometric analysis of IL12B-YFP and IL27A-GFP expression by SILP monocytes (CD11c^{hi} and ^{lo}) and conventional DCs defined as Lin^{-} MHCII^{hi}CD11c^{hi}CD64^{-}CD11b^{low}CD103^{+} (cDC1) and Lin^{-}MHCII^{hi}CD11c^{hi}CD64^{-}CD11b^{+}CD103^{low} (cDC2). Numbers represent the frequency of positive cells. Representative of 3 independent experiments (n=4 mice per group) . The relative proportion of each population among IL27A-GFP+ and IL12B-YFP+ cells in the spleen of infected mice (means from n=4 per group).

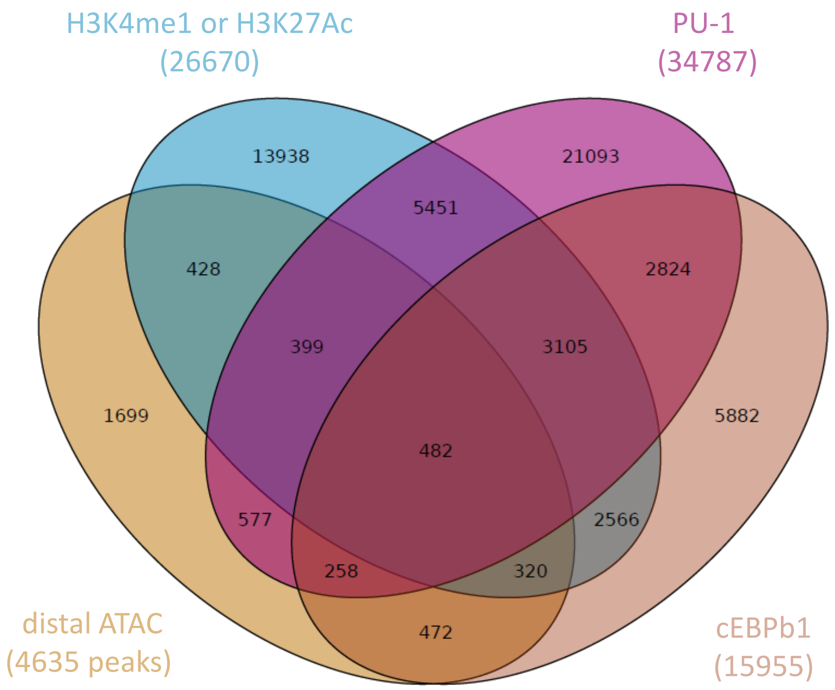

Supplementary figure 4: Venn diagram showing the overlap between distal ATAC peaks (more than 2KB away from known TSS) and public ChIP-Seq data from BMDCs for PU-1, cEBPb1 and H3K4me1 or H3K27Ac histone marks (Garber *et al*, Mol Cell 2012). For PU-1 and cEBPb1, we created atlases (with bedtools suit) that contain the peaks identified in BMDCs in unstimulated and 2h LPS conditions. For Histone marks, we generated an atlas with the peaks identified in LPS-stimulated BMDCs. The total number of peaks for each atlas is indicated. Overlap was performed using Intervene tool (Khan, A. & Mathelier, A. Intervene: a tool for intersection and visualization of multiple gene or genomic region sets. BMC Bioinform. 18, 287 (2017)).

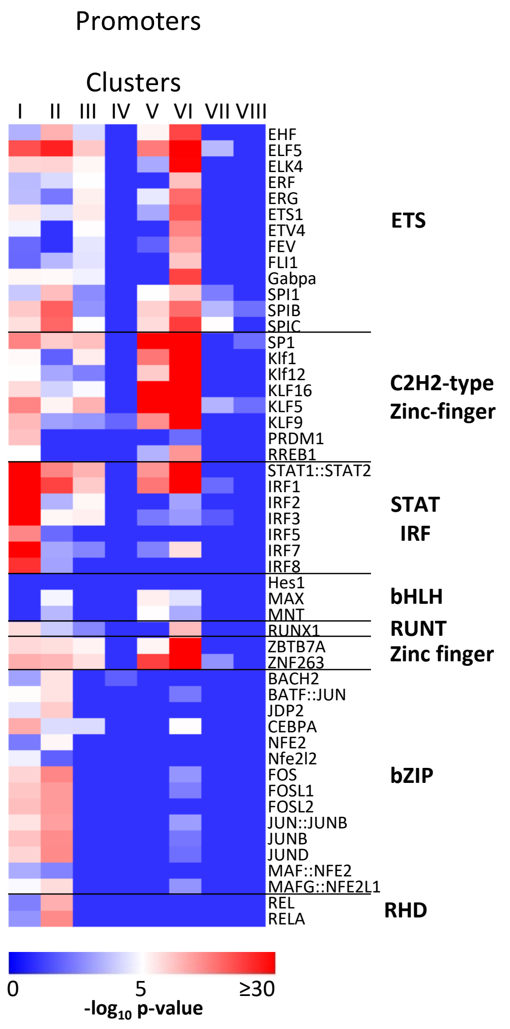

Supplementary figure 5: Motif enrichment analysis for clusters I to VIII at the centers of ATAC peaks located in proximal promoter regions (within 2KB of a TSS) using AME and presented as $-\log_{10}$ of p values. Transcription factors families are shown in the right margin.

Supplementary Figure 6: TNFα signaling does not play a major role in the induction of the second wave of epigenetic remodeling in the periphery

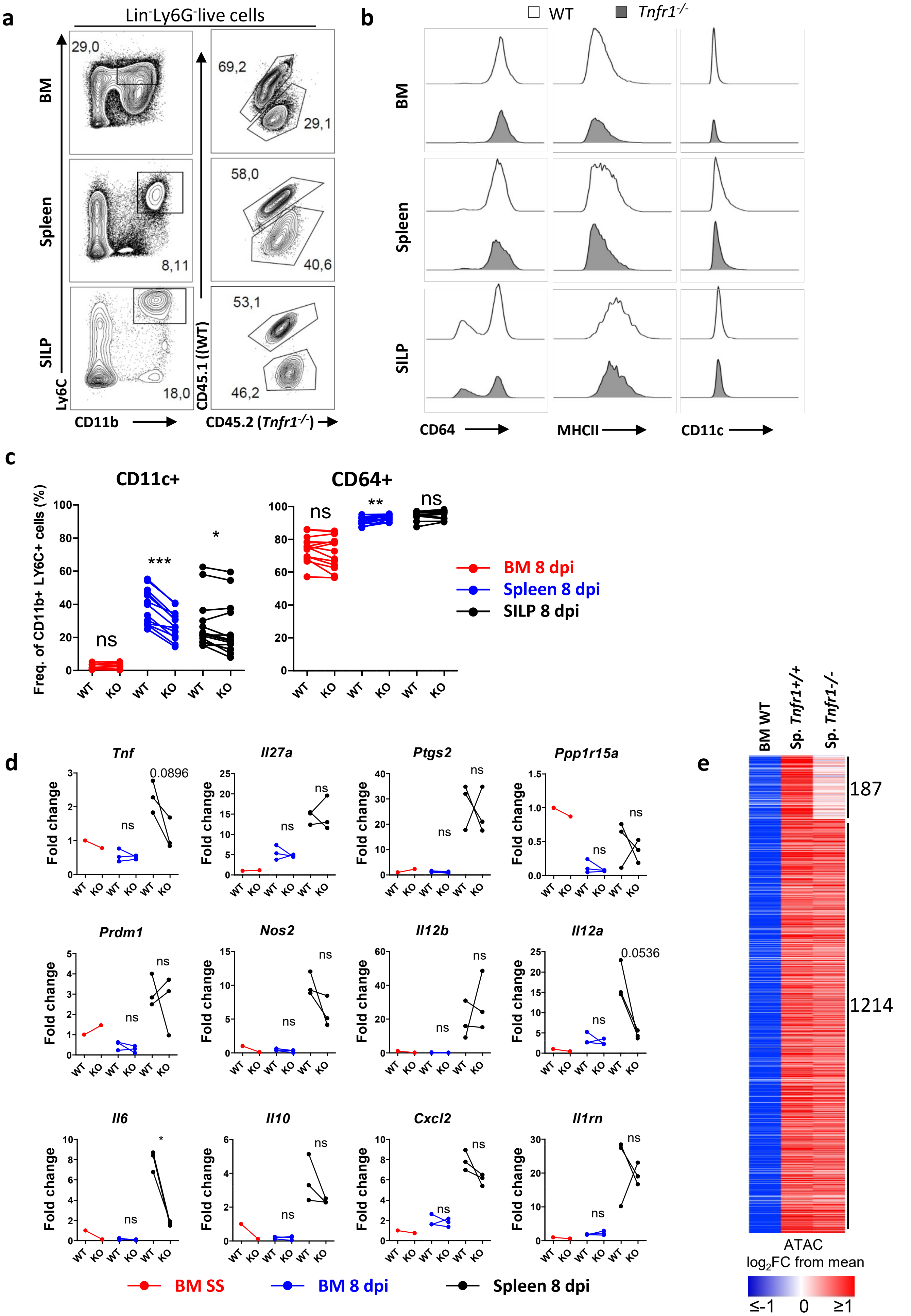

Legend to Supplementary Figure 6: WT/*Tnfr1*$^{-/-}$ mixed bone marrow chimeras were infected per-orally with *T. gondii* cysts. (a) Flow cytometric analysis of CD11b^{+}Ly6C^{+} monocytes from BM, spleen and SILP, of naive (SS) and infected mice (8 dpi). (b) Representative histograms showing expression levels of surface markers and activation markers by CD11b^{+}Ly6C^{+} monocytes from BM, spleen, and SILP of infected mice (8 dpi). (c) Frequency of WT and *Tnfr1*$^{-/-}$ CD11b^{+}Ly6C^{+} monocytes expressing CD64 and CD11c in these organs (each point represents a single mouse, n = 14 mice from two independent experiments). Statistics were calculated using Wilcoxon test. * P < 0.05, ** P < 0.01, *** P<0.001. (d) Histograms showing relative expression level of selected genes by wild-type and *Tnfr1*$^{-/-}$ monocytes from BM and spleen of naïve and infected mice, as assessed by qPCR. The relative expression (fold change) is normalized to the BM of naive mice (each point represents a pool of 3 mice). Statistics were calculated using Wilcoxon test. * P < 0.05, ** P < 0.01. (e) Heatmap showing, among distal regulatory regions from cluster II (as defined in Fig 4), which regions were more or less accessible in CD45.1 wild-type and CD45.2 *Tnfr1*$^{-/-}$ monocytes from the BM and the spleen of infected mice. Out of these 1401 regions, 187 were found to be less accessible in *Tnfr1*$^{-/-}$ cells as compared to their WT counterparts.
Values are represented as Log2 fold-change obtained from the median of each single region.

Supplementary Table 1: List of primers and probes used for single-cell qPCR analysis.

Gene symbol	5'FAMTAMRA3' probe	Forward primer 5'3'	Reverse primer 5'3'
Arg1	TGGTCTCCACCCAGCACCACA	GGCAGAGGTCCAGAAGAATG	ATGCTTCCAACTGCCAGACT
Bach2	CCCTGGCCGTGACCTCCTCA	CACTGGTTGGACAGACGAAA	AACTGTAGCAGTGGCCCAAA
Batf	AAAGCCGACACCCTTCACCTGGA	CCAGAAGAGCCGACAGAGAC	TTCTGTTTCTCCAGGTCCTCAC
Batf3	AGCTGCGTCACCTGAGCGAGG	GAGATTTCGAAGCTGAGGA	TTCATAGGACACAGCAGCAG
Bcl6	CCGCTACAAGGGCAACCTCGC	CGCACAGTGACAAACCATACA	ACCCGTGTGGACAGTCTTGT
Ccl2	CTCAGCCAGATGCAGTTAACGCCCC	CTTCTGGGCCTGCTGTTCA	CCAGCCTACTCATTGGGATCA
Ccl3	CCCGACTGCCTGCTGCTTCTC	AAGTCTTCTCAGCGCCATATG	GTGGAATCTTCCGGCTGTAG
Cebpb	AACTGTCAGCCGGGCCCTGA	GTGTGGACACGGGACTGAC	CCCGCAGGAACATCTTTAAG
Cxcl1	AAGGCAAGCCTCGCGACCAT	CCGAAGTCATAGCCACACTC	TTTCTGAACCAAGGGAGCTT
Cxcl10	TCTCGCAAGGACGGTCCGCTG	GCCGTCATTTTCTGCCTCAT	GCTTCCCTATGGCCCTCATT
Cxcl11	CCGATGCAAAGACAGCGCCC	GATGAACAGGAAGGTCACAGC	GCTTTCTCGATCTCTGCCATT
Cxcl2	CCCACTGCGCCCAGACAGAA	ACATCCAGAGCTTGAGTGTGA	GCCCTTGAGAGTGGCTATG
Cxcl9	CATCAGCACCAGCCGAGGCACG	GAACCCTAGTGATAAGGAATGCA	CTGTTTGAGGTCTTTGAGGGATT
Cxcr3	CCCTGGCCTCTGCAAAGTGGCA	GCTGCTGTCCAGTGGGTTTT	GTTGATGTTGAACAAGGCGC
Ddit3	CTGCCATGACTGCACGTGGACC	CCACACCTGAAAGCAGAACC	ACCGTCTCCAAGGTGAAAGG
Dnajb9	CCGAGAGTGTTTCATACGCTTCTGCA	TGCTGAAGCAAAATTCAGAGA	TCCAATTGTGTCATACTCTTTCC
Ebi3	TCCAGGCTCCCAACTCCACCA	GCCATGCTTCTCGGTATCC	GAGCCTGTAAGTGGCAATGA
Eomes	TCGCTGTGACGGCCTACCAAAACA	CCTTCACCTTCTCAGAGACACAGTT	TCGATCTTTAGCTGGGTGATATCC
Ern1	ACCATCCCAGAATTGGTTCAGGCC	ACGAAGGCCTGACGAAACT	TTTACCCATGTAGAGGATTCCA
Gata3	AGCGAAGGCTGTCGGCAGCA	GCCTGCGGACTCTACCATAA	GCAGGATGTCCCTGCTCTC
Hes1	CTCCTCGCTCACTTCGGACTCCATGT	GGTCCTAACGCAGTGTCACCTT	CAGTGGCCTGAGGCTCTCA
Hmox1	AGAACTTTCAGAAGGGTCAGGTGTCCA	GCCGAGAATGCTGAGTTCAT	AGGAAGCCATCACCAGCTTA
Icam1	TGTCTCCGAGGCCAGGAGCC	AGGGCTGGCATTGTTCTCTAA	GATGGTAGCTGGAAGATCGAA
Id2	CGCCGCTGACCACCCTGAAC	CAGAGACCTGGACAGAACCA	AATTCAGATGCCTGCAAGGA
Ido1	CCCAATGGGCCCATGACATACG	GTCCTGGCAAACTGGAAGAA	CCCACCAGGAAATGAGAACA
Ifit2	CACTGGAGAGCAATCTGCGACAGC	CACAGCAGACAGTTACACAGCA	TGCTATCAGGTTCCAGGTGA
Ifnb1	TGCCTTTGCCATCCAAGAGATGC	TGACGGAGAAGATGCAGAAG	TCCAGGAGACGTACAACAATAGTC
Ifng	TTTGAGGTCAACAACCCACAGGTCCA	GGATGCATTCATGAGTATTGC	GCTTCCTGAGGCTGGATTC
Il10	TCGATGACAGCGCCTCAGCC	TGGGTGAGAAGCTGAAGACC	GCTCCACTGCCTTGCTCTTA
Il12a	TGACATGGTGAAGACGGCCAGA	CTTAGCCAGTCCCGAAACCT	TTGGTCCCGTGTGATGTCT
Il12b	CCCGCCCAAGAACTTGCAGATG	TCAGGGACATCATCAAACCA	AGGGTACTCCCAGCTGACCT
Il23a	CCACAAGGACTCAAGGACAACAGCC	CCCGTATCCAGTGTGAAGATG	CCCTTTGAAGATGTCAGAGTCA
Il27a	TTCCCAATGTTTCCCTGACTTTCCA	ATCTCGATTGCCAGGAGTGA	GTGGTAGCGAGGAAGCAGAGT
Il27ra	AAACTTCTGGCAAACGGGCAGC	CCTTCCAGACGCCATTCTTA	GGGTCCTTCCAGACAAGCA
Il6	CAGAATTGCCATTGCACAACTCTTTTCTCA	GAGGATACCACTCCCAACAGACC	AAGTGCATCATCGTTGTTCATACA
Irf1	TCCGAAGCCGCAACAGACGA	TCGTCAGCAGCAGTCTCTCT	TTCGGCTATCTTCCCTTCCT
Irf2	TCCACGTCCCATCCGTGCC	TGGCTGAACAAGGAGAAGAAG	GAAGAGCGGAGCATCCTTT
Irf3	CCACAAGGACAAGGACGGAGCC	AGCTGCTTCCAGACAGTGG	TTCCATGAAGGCAATCAGATC
Irf4	CTGCCGGCTGCATATCTGCCTGT	GAAGCCTTGGCGCTCTCA	TCACGAGGATGTCCCGGTAA
Irf5	TCTACGAGGTCTGCTCCAACGGC	ATGCCACCTCAGCCGTACA	CATCAGTGGGTTGGCTCTCT
Irf7	TCCAAGCTCCCGGCTAAGTTCG	CCAGTTGATCCGCATAAGGT	TGTGTGGGCAGAGCATTG
Irf8	TTCTACGCCACCCAGAGCCGC	GTGGTGCAGGTCTTTGACAC	CAGGACCACCCTGCTGTC
Itgax	CCTAGCACACGGTTCTCCCTGATGCA	CTGGACTTTGTTAAAGCTGTGATGAG	GACGTGGAGATGAAGTTGTTGAAA
Kdm6b	CTGGCGGCGCTGAACCCTT	AATACCCTGCATGGAGATGC	TGGATATGAATGAGGCATCG
Klf4	TTCCATCCACAGCCGTCCCA	CCCACACAGGTGAGAAACCT	ACGGTAGTGCCTGGTCAGTT
Kmt2a	AAGAACTGCTCATCCTCTCCGCTGC	GCAGTGTTTGGGGAGAGC	TGACTTCTTCATCTGAGCCAAA
Lcn2	TGGACCGCATTGCCTGCCAGGCCCA	GACTTCCGGAGCGATCAGT	ACATCGTAAAGCTGCCTTCTG
MAb21l3	TCATCACCACCCACCACCTGC	GCAGCTGAAGGAGGATGTGT	CAAGTCCAGAAGAGCACGGT
Maf	AACCTGCCACAATCAAGCCTACATCA	CAGAATGAACTAAAGCCACTCC	CACAAGTCACACCCAGAAGG
Map3k11	CTGCAGCCCATCGAGGGTGA	TGCCTGTGATACACCGAGAC	GGGTCTTGTGTTCCATGTCG
Map3k5	CCCTGGGTTTCCTGTGTGCCA	CTCAAGTCCCAGCCCATAGA	TCAGAATCTTCCGTGGTCGT
Nfil3	CCCTCACGGACCAGGGAGCA	GTTACAGCCGCCCTTTCTTT	CAGGAGCCTTTCATGGGTTA
Nos2	CGGGCAGCCTGTGAGACCTTTGA	CAGCTGGGCTGTACAAACCTT	CATTGGAAGTGAAGCGTTTCG
Nr4a1	TTGCAGCCCTCACAGGTGCG	CAATGCTTCGTGTCAGCACT	TACTGTGCGCTTGAAGAAGC
Ppp1r15a	CGCCCACAACTTCTATCTCCTGTCC	GCTTTTGGCAACCAGAACC	GACTGAGCAAGCCCATCAGT
Prdm1	TGCCAGGTCTGCCACAAGAGATTTAGCA	ACACAGGAGAGAAGCCACATGA	GGTGGGTCTTGAGATTGCTTGT
Ptgs2	CCCCCACAGTCAAAGACACTCAGG	CAGGTCATTGGTGGAGAGGT	CTGCAGGTTCTCAGGGATGT
Runx1	CTCTTGCCTCTACCGCTCCGCC	GTAGCGAGATTCAACGACCTC	GACGGTGATGGTCAGAGTGA
Runx3	CGTGAAACTCTTCCCTCGCCCA	CAGGTTCAACGACCTTCGAT	CACTTGGGTAGGGTTGGTGA
S100a6	CGCTTCGCTGTCGACCGTGC	CCCCTCGACCACTCCTTTG	TGACTGATCACTGGGCTAGAAGAA
Scd1	CGCAAACACCCGGCTGTCAA	TTCTTCTCTCACGTGGGTTG	CGGGCTTGTAGTACCTCCTC
Setd1a	CAGGACTCTGCCTACTCCAGCAGCA	CTCAGTCCTCCCAAGGAACC	GGGTTTGAAGGAGGTTGAAGT
Smyd2	ACAACCATGGAGGAGCATTCCAGCT	CGTGGAATGTCAGAAGGAAGA	GCACAGTCTCCGAAGGATTC
Spi1	TTCTCCATCGCTGCCCACGA	ATACCAACGTCCAATGCATG	GTGCGGAGAAATCCCAGTAG
Spib	AGGCCCTCTTGGCTGGCTCC	CTCGGACAGTGAGTCAGACG	GTACAGGCGCAGCTTCTTG
Tbx21	TCATCACTAAGCAAGGACGGCGA	CAAGTTCAACCAGCACCAGA	CCACATCCACAAACATCCTG
Tcf4	TGCAAAGCCGAATCGAAGACCG	GCCTCGTCATCTCCCAATTA	GAATCGCATCGTCCAGTCTT
Tgfb1	CCGAAGCGGACTACTATGCTA	TTTCTCATAGATGGCGTTGTTG	CGCGTGCTAATGGTGGACCG
Tlr3	CCGGGGTGTTTCCAGACAATTGGC	CGAAAGTTGGACTTGTCATCAA	TTCAAGAGGAGGGCGAATAA
Tlr9	CACAGAGCGCAGTTGCCGACT	GCCCAAACTCTCCCTTATGAT	ACCCGCAGCTCGTTATACAC
Tnf	TCGAGTGACAAGCCTGTAGCCCA	CAGACCCTCACACTCAGATCA	CACTTGGTGGTTTGCTACGA
Tnfrsf18	CTGCCAACCAGGCCAGAGGG	GCAAGATCTGCAAGCACTACC	CGGAAGCCAAACACAATATCC
Tsc22d3	CAAGATTGAGCAGGCCATGGACCTC	CAGTGTGGTGGCCCTAGAC	CAGCGTACATCAGGTGGTTC
Usp18	ATGGGAACCACAGATACCGCTGGC	CCTGGAAGGATGTCCAGTGT	TCGTGTAAACCAAGAGATAGGC
Vegfa	CACCATGCAGATCATGCGGATCAAAC	GCTGTGCAGGCTGCTGTAAC	AGCTCATCTCTCCTATGTGCTG
Xbp1	CTGAGTCCGCAGCAGGTGCA	GGAGTGGAGTAAGGCTGGTG	GGCAACAGTGTCAGAGTCCA
Zbtb20	CCGAGCGCATTCACAGCATCA	ACACCGGGTCATCTGATTGT	AGCACGGAATTGCTGAAGTT
Zfp36	CCAAGTGCCAGTTTGCTCACGGC	CTCAGAAAGCGGGCGTTGT	GATTGGCTTGGCGAAGTTCA
Zmynd11	TCTTAATGCTCCTGCAAACTGGGCA	GAGTTCAGGCTTAGAGACAGCAG	TCAAGTAGGTGCCCATTTCC

Samples	# reads
ATAC_Mono_BM_SteadyState_rep1	20972376
ATAC_Mono_BM_SteadyState_rep2	90529134
ATAC_Mono_Spleen_SteadyState_rep1	16892826
ATAC_Mono_Spleen_SteadyState_rep2	22387344
ATAC_Mono_BM_Toxo_rep1	23277502
ATAC_Mono_WT_BM_Toxo_rep2	23083388
ATAC_Mono_Spleen_Toxo_rep1	22802296
ATAC_Mono_WT_Spleen_Toxo_rep2	21312520
ATAC_MonoHigh_Spleen_Toxo	14679516
ATAC_MonoLow_Spleen_Toxo	13997642
ATAC_Mono_LP_Toxo_rep1	18967388
ATAC_Mono_LP_Toxo_rep2	72264194
ATAC_Mono_STAT1-KO_BM_Toxo	23270646
ATAC_Mono_STAT1-KO_Spleen_Toxo	26205626

Supplementary Table 2: List of ATAC-seq samples.

BMDCs	# peaks	
	Resting	LPS 2h
STAT1	254	4985
IRF1	1747	10419
PU-1	29988	27406
JUNB	6323	9485
RELA	385	13179
CEBPb	6261	15261

BMMs	# peaks	
	Resting	IFNg 3h
IRF8	6230	5741
IRF1	2338	14196

Supplementary Table 3: Number of ChIP-seq peaks of resting or LPS-stimulated BMDCs (GSE36099) and IFNγ-stimulated BMMs (GSE77886) for the indicated transcription factors.
