# Supplementary material for: Monocytes undergo multi-step differentiation in mice during oral infection by Toxoplasma gondii
Source: Commun Biol. 2019 Dec 18;2:472. doi: 10.1038/s42003-019-0718-6 (PMC6920430; doi:10.1038/s42003-019-0718-6)
Supplement: Supplementary file 2 — Description of Additional Supplementary Files [file 42003_2019_718_MOESM2_ESM.docx]

**Description of additional supplementary files**

**Supplementary Data 1**: List of the ATAC peaks identified in clusters I to VIII (related to Figure 4). For each region, the associated gene is indicated and the quantification in each sample is shown.

**Supplementary Data 2:** Raw data supporting graphs and charts presented in the figures.
