# Supplementary material for: Monocytes undergo multi-step differentiation in mice during oral infection by Toxoplasma gondii
Source: Commun Biol. 2019 Dec 18;2:472. doi: 10.1038/s42003-019-0718-6 (PMC6920430; doi:10.1038/s42003-019-0718-6)
Supplement: Supplementary file 5 — Reporting Summary [file 42003_2019_718_MOESM5_ESM.pdf]

nature research

Corresponding author(s): Stanislas GORIELY

Last updated by author(s): Oct 10, 2019

Reporting Summary

Nature Research wishes to improve the reproducibility of the work that we publish. This form provides structure for consistency and transparency in reporting. For further information on Nature Research policies, see Authors & Referees and the Editorial Policy Checklist.

Statistics

For all statistical analyses, confirm that the following items are present in the figure legend, table legend, main text, or Methods section.

n/a	Confirmed	
☐	☒	The exact sample size (*n*) for each experimental group/condition, given as a discrete number and unit of measurement
☐	☒	A statement on whether measurements were taken from distinct samples or whether the same sample was measured repeatedly
☐	☒	The statistical test(s) used AND whether they are one- or two-sided<br>*Only common tests should be described solely by name; describe more complex techniques in the Methods section.*
☒	☐	A description of all covariates tested
☒	☐	A description of any assumptions or corrections, such as tests of normality and adjustment for multiple comparisons
☐	☒	A full description of the statistical parameters including central tendency (e.g. means) or other basic estimates (e.g. regression coefficient) AND variation (e.g. standard deviation) or associated estimates of uncertainty (e.g. confidence intervals)
☒	☐	For null hypothesis testing, the test statistic (e.g. *F*, *t*, *r*) with confidence intervals, effect sizes, degrees of freedom and *P* value noted<br>*Give P values as exact values whenever suitable.*
☒	☐	For Bayesian analysis, information on the choice of priors and Markov chain Monte Carlo settings
☒	☐	For hierarchical and complex designs, identification of the appropriate level for tests and full reporting of outcomes
☒	☐	Estimates of effect sizes (e.g. Cohen's *d*, Pearson's *r*), indicating how they were calculated

Our web collection on statistics for biologists contains articles on many of the points above.

Software and code

Policy information about availability of computer code

Data collection	Flow cytometry data were acquired on a BD LSRFortessa™ cell analyzer after successfully running Cytometer Setup and Tracking beads (BD) and using the application settings featured on FACSDiva™ software (v 6.2).<br>Cells were sorted on a BD FACSAria™ III.<br>Single cell qPCR data were collected on a Fluidigm Biomark HD.
Data analysis	Flow cytometry data were analyzed using the FlowJo v10 software (Tree Star) and the and the t-Distributed Stochastic Neighbor Embedding (tSNE) plugin.<br>Single-cell PCR data were collected and analyzed using Fluidigm Real-time PCR analysis software (version 4.3.1) and Fluidigm Singular Analysis Toolset Software. Heatmap of expression data and violin plots were generated in Singular Analysis Toolset Software. PCA was performed in "R" using ggplot2 package (H. Wickham. ggplot2: Elegant Graphics for Data Analysis. Springer-Verlag New York, 2016).<br>Prism 6.0 was used for statistical analysis. Mann-Whitney test was used to compare two data sets. For all analyses, no data points were excluded.<br>Seqmonk was used to analyse ATAC-seq data and overlap ChIP-seq data.<br>usegalaxy.org/deeptool suit was used to generate FrIP normalized bigwig files.<br>Genomic Regions Enrichment of Annotations Tool (GREAT) was used to analyse the functional significance of ATAC-seq differential regions and for gene set enrichment analysis.<br>Meme-suit was used for motif analysis.<br>MeV (Multiple Experiment Viewer) was used to generate the heatmaps.<br>IGV browser (Broad institute) was used to visualise the normalised bigwig files.

For manuscripts utilizing custom algorithms or software that are central to the research but not yet described in published literature, software must be made available to editors/reviewers. We strongly encourage code deposition in a community repository (e.g. GitHub). See the Nature Research guidelines for submitting code & software for further information.

Data

Policy information about availability of data

All manuscripts must include a data availability statement. This statement should provide the following information, where applicable:

- Accession codes, unique identifiers, or web links for publicly available datasets
- A list of figures that have associated raw data
- A description of any restrictions on data availability

ATAC-Seq data that support the findings reported in this study have been deposited in the GEO Repository with the accession code GSE129724.

Field-specific reporting

Please select the one below that is the best fit for your research. If you are not sure, read the appropriate sections before making your selection.

☒ Life sciences ☐ Behavioural & social sciences ☐ Ecological, evolutionary & environmental sciences

For a reference copy of the document with all sections, see nature.com/documents/nr-reporting-summary-flat.pdf

Life sciences study design

All studies must disclose on these points even when the disclosure is negative.

Sample size	Sample size was determined according to standard practice in the field. For FACS data, 4 to 10 mice per group were included. ATAC-seq samples were generated from cells sorted from pools of 3 to 7 mice. For single cell qPCR, 48, 64 or 89 individual monocytes were sorted (depending on the group). No statistical methods were used to predetermine sample sizes.
Data exclusions	No data points were excluded from analyses. All individual data points are shown in bar plot graphs.
Replication	All data presented are from representative independent experiments that were not pooled with other similar experiments.<br>Figure 1a: Experiment was performed once on 5 independent mice for each timepoint.<br>Figure 1b: Data are representative of three experiments (n = 10 mice for each experiment)<br>Figure 1c: Histograms show expression levels of surface markers monocytes. Numbers indicate percentage of positive cells, expressed as mean±SD from 10 individual mice.<br>Figure 1d to g: Unsupervised analysis of single events from concatenation of one naive and one infected mouse.<br>Figure 2 and 3a: Single-cell qPCR was performed on 603 individual cells isolated from 3 pooled naive or 7 pooled infected mice.<br>Figure 3b: Data are representative of at least 3 independent experiments (n = 4 mice per group). Histograms show frequency of positive cells . Horizontal bars indicate median±interquartile range and are representative of more than three experiments.<br>Figure 3c: Histograms indicate percentage of positive cells for one representative mouse (n=4).<br>Figure 3d: Relative proportion of each population as means from n=4 per group.<br>Figure 4 et 5a, b, c: ATAC-seq samples were generated from cells sorted from pools of 3 mice.<br>Figure 5d: Histograms indicate percentage of positive cells for one representative mouse (Left). Histogram shows frequency of double positive cells (n = 5 mice) (right). The experiment was performed once on five independent mice.<br>Figure 6a and b: Histograms indicate percentage of positive cells for one representative mouse (n=6 mice).<br>figure 6C: Histograms indicate percentage of positive cells (n=6 mice, each point represents a single mouse).<br>Figure 6D: Single-cell qPCR was performed on 48 individual monocytes for each experimental group from 3 pooled naive or 7 pooled infected mice.<br>Figure 7: ATAC-seq data were generated from a pool of 7 mice.
Randomization	Mice were grouped according to genotype. No randomization was used to attribute treatments in in vivo studies.
Blinding	No blinding was applied in this study.

Reporting for specific materials, systems and methods

We require information from authors about some types of materials, experimental systems and methods used in many studies. Here, indicate whether each material, system or method listed is relevant to your study. If you are not sure if a list item applies to your research, read the appropriate section before selecting a response.

Materials & experimental systems

n/a	Involved in the study
☐	☒ Antibodies
☐	☒ Eukaryotic cell lines
☒	☐ Palaeontology
☐	☒ Animals and other organisms
☒	☐ Human research participants
☒	☐ Clinical data

Methods

n/a	Involved in the study
☐	☒ ChIP-seq
☐	☒ Flow cytometry
☒	☐ MRI-based neuroimaging

Antibodies

Antibodies used	All FACS antibodies were anti-mouse showing a validated anti-mouse reactivity (clone; fluorochrome):<br>- from Life Technologies: Live/Dead Fixable Aqua Dead Cell Stain (used at a 1:1000 dilution).<br>- from BD: CD3 (145-2C11; APC-Cy7), CD19 (1D3; APC-Cy7), NK1.1 (PK136; APC-Cy7), Ly6G (1A8; PerCP-Cy5), LY6G (1A8; APC-Cy7), Ly6C (AL-21; BV421), CD11b (M1/70; AF700), CD11c (HL3; PE-Cy7), CD8a (53.6.7; PerCP), CD64 a and b alloantigens (X54-5/7.1; AF647), CD64 a and b alloantigens (X54-5/7.1;BV785), CD40 (3/23; BV711), CD80 (16-10A1; PE), CD86 (GL1; APC), CD45.1 (A20; Pe-Cy7). Each antibody was used at a 1:200 dilution.<br>-from eBiosciences: MHCII (IA/IE) (M5/114.15.2; FITC), c-Kit (CD117) (2B8; FITC), CD45.2 (104; PerCP-Cy5). Each antibody was used at a 1:200 dilution.<br>- from Cell signaling Technology: C-Jun (60A8; PE) and phospho-c-Jun (Ser73) (D47G9 XP; Alexa Fluor 488).These antibodies were used following manufacturer's instruction.
Validation	Antibodies were validated by the manufacturer. For some FACS antibodies, optimal dilution was determined by titration and based on observed staining indexes.

Eukaryotic cell lines

Policy information about cell lines

Cell line source(s)	Human primary fibroblasts (ATCC number: CCL-186; IMR-90)
Authentication	N/A
Mycoplasma contamination	Not tested
Commonly misidentified lines<br>(See ICLAC register)	N/A

Animals and other organisms

Policy information about studies involving animals; ARRIVE guidelines recommended for reporting animal research

Laboratory animals	C57BL/6 mice were purchased from Envigo. Yet40 (p40-IRES-eYFP, B6.129-Il12btm1Lky/J) and Ly5.1 congenic mice on C57BL/6 background were obtained from the Jackson Laboratory. Stat1-/- on C57BL/6 background were kindly provided by D.E. Levy (New York University School of Medicine, NYC, USA). For IL27A reporter mice, BAC clones containing Il27a chromosomal regions (BAC ID: RP24-15802) were obtained from the Children's Hospital of Oakland Research Institute BACPAC Resource Center, and then modified so as to insert eGFP gene within first exon of Il27a gene by homologous recombination (Cyagen Biosciences). The resulting modified BAC was then micro-injected into the pronuclei of C57BL/6 fertilized eggs and transferred into the oviducts of pseudo-pregnant females. The pups were screened for the transgene sequence by quantitative real-time PCR using the following oligos: FAM-TTCAAGTCCGCCATGCCCGAATAMRA, 5'-CCACATGAAGCAGGACTT-3' and 5'-GGTGCGCTCCTGGACGTA-3'. One of the transgene-bearing pups was subsequently bred with C57BL/6 mice to generate Il27a eGFP reporter mice and littermate controls. All experiments were performed on sex- and age-matched (8 to 12 weeks of age) mice. All animal studies were approved by the Animal Welfare and Ethics Committee of the ULB-IBMM. All experiments were conducted in accordance with the recommended guidelines and regulations.
Wild animals	None were used in this study.
Field-collected samples	N/A
Ethics oversight	All animal work was carried out in compliance with and after approval by the institutional Animal Care and local committee for animal welfare.

Note that full information on the approval of the study protocol must also be provided in the manuscript.

ChIP-seq

Data deposition

☒ Confirm that both raw and final processed data have been deposited in a public database such as GEO.

☒ Confirm that you have deposited or provided access to graph files (e.g. BED files) for the called peaks.

Data access links<br>*May remain private before publication.*	https://www.ncbi.nlm.nih.gov/geo/query/acc.cgi?acc=GSE129724<br>Access code: mxaxkumwrbkffkt
Files in database submission	GSE129724 Multi-step Epigenetic Programming of Monocytes during Infection<br>Apr 11, 2020 approved None<br>GSM3720846 ATAC_MonoLow_Spleen_Toxo Apr 11, 2020 approved BIGWIG NARROWPEAK<br>GSM3720847 ATAC_MonoHigh_Spleen_Toxo Apr 11, 2020 approved BIGWIG NARROWPEAK<br>GSM3720848 ATAC_Mono_Spleen_SteadyState_rep1 Apr 11, 2020 approved BIGWIG NARROWPEAK<br>GSM3720849 ATAC_Mono_LP_Toxo Apr 11, 2020 approved BIGWIG NARROWPEAK<br>GSM3720850 ATAC_Mono_Spleen_Toxo Apr 11, 2020 approved BIGWIG NARROWPEAK<br>GSM3720851 ATAC_Mono_BM_Toxo Apr 11, 2020 approved BIGWIG NARROWPEAK<br>GSM3720852 ATAC_Mono_Spleen_SteadyState_rep2 Apr 11, 2020 approved BIGWIG NARROWPEAK<br>GSM3720853 ATAC_Mono_BM_SteadyState Apr 11, 2020 approved BIGWIG NARROWPEAK<br>GSM3720854 ATAC_Mono_WT_Spleen_Toxo Apr 11, 2020 approved BIGWIG NARROWPEAK<br>GSM3720855 ATAC_Mono_STAT1-KO_Spleen_Toxo Apr 11, 2020 approved BIGWIG<br>NARROWPEAK<br>GSM3720856 ATAC_Mono_WT_BM_Toxo Apr 11, 2020 approved BIGWIG NARROWPEAK<br>GSM3720857 ATAC_Mono_STAT1-KO_BM_Toxo Apr 11, 2020 approved BIGWIG NARROWPEAK<br>--
Genome browser session<br>(e.g. UCSC)	no longer applicable

Methodology

Replicates	ATAC-seq samples were prepared from cells isolated from pools of 3 to 7 animals and processed as individual samples.
Sequencing depth	All NGS experiments have a sequencing depth between 13-90 millions mapped reads
Antibodies	N/A
Peak calling parameters	Paired-end reads were mapped to mouse genome mm10 with Bowtie2 using default parameters for paired-end reads. Peaks were called with MACS2 using the following parameters: -f BAMPE -g mm -q 0.05 --nomodel --call-summits -B –SPMR.
Data quality	Reads that mapped several regions, or with insufficient mapping quality, were removed with samtools view. We also removed reads located within the blacklist of the ENCODE project. Duplicate reads were removed with MarkDuplicates tools (Picard suite)
Software	Bowtie2 was used for mapping.<br>Picard suite was used to remove duplicates.<br>MACS2 was used for peak calling.

Flow Cytometry

Plots

Confirm that:

- [x] The axis labels state the marker and fluorochrome used (e.g. CD4-FITC).
- [x] The axis scales are clearly visible. Include numbers along axes only for bottom left plot of group (a 'group' is an analysis of identical markers).
- [x] All plots are contour plots with outliers or pseudocolor plots.
- [x] A numerical value for number of cells or percentage (with statistics) is provided.

Methodology

Sample preparation	Single-cell suspensions were washed in PBS and incubated in mAb 2.4G2 (BD Biosciences) and fluorescently conjugated antibodies for cell surface markers in PBS for 20 minutes at 4°C in the dark. Cells were washed and resuspended in PBS 2%(vol/vol) fetal calf serum (FCS).<br>For c-Jun and Phospho-c-Jun staining: after extracellular staining, cells were washed in PBS and fixed in 1 ml paraformaldehyde 4% for 15 minutes at 37°C. Cells were washed in PBS and permeabilized by adding ice-cold methanol 90% while gently vortexing and incubated 30 minutes on ice in the dark. Cells were washed twice to remove methanol and incubated in PBS BSA 0.5% and C-Jun 60A8 rabbit mAb PE and phospho-c-Jun (Ser73) D47G9 XP rabbit mAb Alexa Fluor 488 (Cell signaling Technology) for 30 minutes on ice in the dark. Cells were washed and resuspended in PBS BSA 0.5%.
Instrument	Cells were sorted on a BD FACSAria™ III using fluorescence minus one (FMO) controls (see Supplementary Fig. S2 or gating strategies). Flow cytometry data were acquired on a BD LSRFortessa™ cell analyzer after successfully running Cytometer Setup and Tracking beads (BD).
Software	The FACSDiva™ software (v 6.2) was used to collect the data. Data were analyzed using FlowJo v10 software (Tree Star) and the t-Distributed Stochastic Neighbor Embedding (tSNE) plugin.
Cell population abundance	A minimum of 1000 target cells were recorded per replicate.
Gating strategy	A large gate was set on a FSC-A/SSC-A, and singlets were selected on an FSC-A/FSC-H plot , then on an SSC-A/SSC-H plot. Dead cells were eliminated on a Live Dead/Cd11b plot. Detailed gating strategies for cell sorting are shown in Supplementary Fig. S2 For FACS data, lineage-negative cells were selected based on a lineage (Gr1, NK1.1, CD19) / CD11b plot. Granulocytes were excluded based on LY6G expression. Monocytes were gated as CD11b+ LY6C+ cells.

- [x] Tick this box to confirm that a figure exemplifying the gating strategy is provided in the Supplementary Information.
